# Supplementary material for: ePOCT+ and the medAL-suite: Development of an electronic clinical decision support algorithm and digital platform for pediatric outpatients in low- and middle-income countries
Source: PLOS Digit Health. 2023 Jan 19;2(1):e0000170. doi: 10.1371/journal.pdig.0000170 (PMC9931356; doi:10.1371/journal.pdig.0000170)
Supplement: S1 Appendix — (DOCX) [file pdig.0000170.s001.docx]

**S1 Appendix: Prevalence of specific symptoms and diagnoses not covered in IMCI from Tanzania**

**Table A:** Notable symptoms and diagnoses for children above 2 months not existing in IMCI added to ePOCT+

| **Additional non-IMCI conditions** | **Frequencies from Tanzanian studies/databases** |
| --- | --- |
| ***Symptoms*** | |
| Headache | - 34% (0-5 years) and 81% (5-17 years) of febrile outpatients[1] |
| Abdominal pain | - 13% (0-5 years) and 23% (5-14 years) of febrile outpatients[1]  - 4.6% (2 months – 5 years) of febrile outpatients[2] |
| Sore throat | - 13% (5-14 years) of febrile outpatients[1] |
| Dental pain | - 30.2% dental pain among children age 12 to 19 years old[3] |
| ***Diagnoses / Classifications*** | |
| Fever without source (Undifferentiated febrile illness) | - 6.2% (0-5 years) and 7.1% (5-17 years) of outpatients[4] |
| Urinary tract infection | - 5.9% (2 months – 10 years) of febrile outpatients[5]  - 19% (0-5 years) and 16% (5-14 years) of febrile outpatients[1]  - 9.9% (0-5 years) and 19.7% (5-17 years) of outpatients[4]  - 8.1% (1 month – 5 years) of all outpatients[6]  - 18.6% (2-13 years) of febrile outpatients[7] |
| Eye disease | - 2% (1 month – 5 years) of all outpatients[6] |
| Trauma including burns | - 4.3% (0-5 years) and 21.6% (5-17 years) of outpatients[4]  - 9.7% (all ages) of outpatients[8] |
| Sexually Transmitted Infections | - 0.1 to 13.7% prevalence among adolescents 12 to 19 years old[9] |
| Dental caries and related oral problems | - 19.2% dental caries, 45.3% perceived need for dental care: prevalence among children age 13-19 years[3]  - 30.7% dental caries: prevalence among all age groups[10] |

**References**

1. Hercik C, Cosmas L, Mogeni OD, Wamola N, Kohi W, Omballa V, et al. A diagnostic and epidemiologic investigation of acute febrile illness (AFI) in Kilombero, Tanzania. PLoS One. 2017;12(12):e0189712-e. doi: 10.1371/journal.pone.0189712. PubMed PMID: 29287070.

2. Keitel K, Kagoro F, Samaka J, Masimba J, Said Z, Temba H, et al. A novel electronic algorithm using host biomarker point-of-care tests for the management of febrile illnesses in Tanzanian children (e-POCT): A randomized, controlled non-inferiority trial. PLoS medicine. 2017;14(10):e1002411. Epub 2017/10/24. doi: 10.1371/journal.pmed.1002411. PubMed PMID: 29059253; PubMed Central PMCID: PMCPMC5653205.

3. Mashoto KO, Astrøm AN, David J, Masalu JR. Dental pain, oral impacts and perceived need for dental treatment in Tanzanian school students: a cross-sectional study. Health Qual Life Outcomes. 2009;7:73-. doi: 10.1186/1477-7525-7-73. PubMed PMID: 19643004.

4. McHomvu E, Mbunda G, Simon N, Kitila F, Temba Y, Msumba I, et al. Diagnoses made in an Emergency Department in rural sub-Saharan Africa. Swiss Med Wkly. 2019;149:w20018. Epub 2019/02/05. doi: 10.4414/smw.2019.20018. PubMed PMID: 30715723.

5. D'Acremont V, Kilowoko M, Kyungu E, Philipina S, Sangu W, Kahama-Maro J, et al. Beyond malaria--causes of fever in outpatient Tanzanian children. The New England journal of medicine. 2014;370(9):809-17. Epub 2014/02/28. doi: 10.1056/NEJMoa1214482. PubMed PMID: 24571753.

6. MoHCDGEC-HMIS Unit. [cited 2020 11.02.2020]. 2019:[District Health Information 2 (DHIS ) Tanzania]. Available from: <http://dhis.moh.go.tz/dhis-web-pivot/app/index.html>].

7. Chipwaza B, Mhamphi GG, Ngatunga SD, Selemani M, Amuri M, Mugasa JP, et al. Prevalence of Bacterial Febrile Illnesses in Children in Kilosa District, Tanzania. Plos Neglected Tropical Diseases. 2015;9(5). doi: 10.1371/journal.pntd.0003750. PubMed PMID: WOS:000355303600020.

8. Sawe HR, Mfinanga JA, Mbaya KR, Koka PM, Kilindimo SS, Runyon MS, et al. Trauma burden in Tanzania: a one-day survey of all district and regional public hospitals. BMC Emergency Medicine. 2017;17(1):30. doi: 10.1186/s12873-017-0141-6.

9. Nkata H, Teixeira R, Barros H. A scoping review on sexual and reproductive health behaviors among Tanzanian adolescents. Public Health Reviews. 2019;40(1):4. doi: 10.1186/s40985-019-0114-2.

10. Teshome A, Muche A, Girma B. Prevalence of Dental Caries and Associated Factors in East Africa, 2000-2020: Systematic Review and Meta-Analysis. Front Public Health. 2021;9:645091-. doi: 10.3389/fpubh.2021.645091. PubMed PMID: 33996722.
