# Supplementary material for: ePOCT+ and the medAL-suite: Development of an electronic clinical decision support algorithm and digital platform for pediatric outpatients in low- and middle-income countries
Source: PLOS Digit Health. 2023 Jan 19;2(1):e0000170. doi: 10.1371/journal.pdig.0000170 (PMC9931356; doi:10.1371/journal.pdig.0000170)
Supplement: S2 Appendix — (DOCX) [file pdig.0000170.s002.docx]

**S2 Appendix: Delphi survey on the reliability and feasibility of measurement of symptoms and signs**

**Methods**

A pre-selection of clinical elements were identified based on a systematic review on triage tools (ETAT, PEWS, pSATS, ESI, TOPRS, IMCI) [1]. Symptoms and signs were excluded from the Delphi survey if a) the quality or predictive value was insufficient based on previous research [2], b) they are collected anyway during registration of the patient or c) they were known to be unfeasible for triage in Tanzanian primary care beforehand (for example laboratory tests not available at primary care health facilities). The Delphi survey was based on a recent Delphi study among international experts on predictors of sepsis in children under five [2] and included questions about each clinical element based on three domains: 1. Reliability of measurement, 2. Frequency of finding an abnormal value, and 3. Level of training required. Additionally, availability of instruments to measure vital signs and other challenges in collecting each element were evaluated. The answers were classified using a 5-point Likert scale: minimal, moderate, high, not applicable, I don’t know. The answer options for the availability of vital sign instruments were yes/no/I don’t know. We also collected data on the professional background and expertise of the participants.

**Analysis**

We analysed the results of the Delphi study according to the three domains. The answers were classified into a score 0-3: 0 = not applicable, and 1 – 3 for increasing strength of the answer. We calculated the total score of all participants per variable for each domain, resulting in a sum score of 0 - 90, stratified per level of care (dispensary or health centre). We also calculated the maximum score per variable, excluding the participants who answered ‘I don’t know’ to that particular variable. To facilitate comparison across items, we calculated the sum score as a percentage of the maximum score. We also created a composite sum score per item: sum score of domain 1 + score domain 2 – score domain 3, divided by the sum of max scores of all three domains, and separated for dispensaries and health centers. We did not predefine a threshold for inclusion in the final proposed triage tool. Analyses were performed in SPSS (version 25.0).

**Results**

The results of the Delphi survey (number of participants=30) are shown in Table 2. While most signs and symptoms were feasible to assess at primary care health facilities, ‘capillary refill time’, ‘pain score (0-10)’, ‘assessment of cold skin’, and ‘weak and fast pulse’ had a lower score and were excluded from ePOCT+. Vital signs and anthropometric measurements including MUAC, heart rate and oxygen saturation also had a lower score due to lack of instruments and need for training.

**Table A: Composite score on different domains per triage item**

|  | **Combined score dispensary** | | | **Combined score health centre** | | |
| --- | --- | --- | --- | --- | --- | --- |
|  |  |  |  |  |  |  |
|  | *combined sum (domain 1+2-3)* | *combined max (domain 1+2+3)* | *%* | *combined sum (domain 1+2-3)* | *combined max (domain 1+2+3)* | *%* |
| General / past medical history* |  |  |  |  |  |  |
| Urgent referral status | 43 | 216 | 20% | 53 | 231 | 23% |
| Measurements / vital signs |  |  |  |  |  |  |
| MUAC (mm) | 7 | 195 | 4% | 16 | 207 | 8% |
| Temperature | 88 | 237 | 37% | 81 | 243 | 33% |
| Heart rate (HR) | 24 | 189 | 13% | 23 | 198 | 12% |
| Respiratory rate (RR) | 46 | 216 | 21% | 43 | 219 | 20% |
| Oxygen saturation (SpO2) | -10 | 159 | -6% | 7 | 174 | 4% |
| Pain score (0 - 10) | -4 | 150 | -3% | -7 | 153 | -5% |
| Weight | 80 | 234 | 34% | 73 | 234 | 31% |
| Capillary Refill Time | 10 | 162 | 6% | 12 | 180 | 7% |
| Airway / breathing |  |  |  |  |  |  |
| Central cyanosis / is the child blue? | 52 | 216 | 24% | 52 | 225 | 23% |
| Apnea (observed or reported) | 54 | 228 | 24% | 54 | 231 | 23% |
| Difficulty breathing (reported) | 73 | 240 | 30% | 77 | 249 | 31% |
| Difficulty breathing (observed: chest indrawing, grunting, nasal flaring) | 73 | 246 | 30% | 67 | 249 | 27% |
| Fast breathing (reported) | 79 | 240 | 33% | 75 | 243 | 31% |
| Circulation |  |  |  |  |  |  |
| Skin cold (cool peripheries) | 31 | 207 | 15% | 34 | 225 | 15% |
| Weak and fast pulse | 40 | 216 | 19% | 30 | 213 | 14% |
| Pallor - palmar, oral, conjunctival | 77 | 237 | 32% | 75 | 240 | 31% |
| Neurological |  |  |  |  |  |  |
| Irritability, restlessness | 87 | 237 | 37% | 84 | 240 | 35% |
| Convulsions (reported, history of) | 77 | 243 | 32% | 73 | 249 | 29% |
| Convulsing now, actively | 86 | 243 | 35% | 80 | 243 | 33% |
| Not able to drink or feed anything | 84 | 243 | 35% | 81 | 246 | 33% |
| Lethargy (AVPU) | 50 | 219 | 23% | 53 | 231 | 23% |
| Mobility - unable to move as normal | 41 | 222 | 18% | 37 | 222 | 17% |
| Dehydration |  |  |  |  |  |  |
| Sunken eyes | 77 | 243 | 32% | 68 | 240 | 28% |
| Reduced urine production | 47 | 210 | 22% | 43 | 210 | 20% |
| Infection |  |  |  |  |  |  |
| Fever (reported) | 103 | 234 | 44% | 95 | 240 | 40% |
| Gastrointestinal |  |  |  |  |  |  |
| Diarrhea | 75 | 237 | 32% | 76 | 240 | 32% |
| Vomiting everything | 87 | 240 | 36% | 81 | 240 | 34% |
| Trauma |  |  |  |  |  |  |
| Significant trauma or other urgent surgical condition | 26 | 207 | 13% | 22 | 216 | 10% |
| Burns | 35 | 216 | 16% | 47 | 231 | 20% |
| Poisoning | 39 | 213 | 18% | 37 | 216 | 17% |
| Severe pain | 81 | 234 | 35% | 75 | 228 | 33% |
| **Average score overall** |  |  | **24%** |  |  | **23%** |

Footnote:

Colours = heat map per level of care, ranged from lowest % of maximum score to highest % of maximum score. Green = above average, red = below average, white = average.

*For duration of illness, number of previous admissions, admitted in past 2 days and history of HIV/sickle cell/palsy information was not available for all domains, so were left out of the composite score.

**References**

1. Hansoti B, Jenson A, Keefe D, De Ramirez SS, Anest T, Twomey M, et al. Reliability and validity of pediatric triage tools evaluated in Low resource settings: a systematic review. BMC Pediatrics. 2017;17(1):37. doi: 10.1186/s12887-017-0796-x.

2. Fung JST, Akech S, Kissoon N, Wiens MO, English M, Ansermino JM. Determining predictors of sepsis at triage among children under 5 years of age in resource-limited settings: A modified Delphi process. PLoS One. 2019;14(1):e0211274. Epub 2019/01/29. doi: 10.1371/journal.pone.0211274. PubMed PMID: 30689660; PubMed Central PMCID: PMCPMC6349330.
