# Supplementary material for: ePOCT+ and the medAL-suite: Development of an electronic clinical decision support algorithm and digital platform for pediatric outpatients in low- and middle-income countries
Source: PLOS Digit Health. 2023 Jan 19;2(1):e0000170. doi: 10.1371/journal.pdig.0000170 (PMC9931356; doi:10.1371/journal.pdig.0000170)
Supplement: S3 Appendix — (DOCX) [file pdig.0000170.s003.docx]

**S3 Appendix: Prognostic value of predictors used in the ePOCT and ALMANACH electronic clinical decision support algorithms**

**Methods**

This is a sub-analysis of previously published data from the ePOCT study [1]. Briefly, a randomized controlled non-inferiority study was performed among children aged 2-59 months presenting with an acute febrile illness to 9 outpatient clinics in Dar es Salaam, Tanzania between December 2014 and February 2016. Patients were randomized by block to receive care using ALMANACH or ePOCT, a first- and a second-generation electronic clinical decision support algorithm (CDSA). The prognostic outcome for the present analysis was clinical failure by day 7. Symptoms, signs and tests were measured/assessed by study clinicians prompted by the respective eCDSAs.

**Analysis**

A bivariate logistic regression analysis and descriptive statistics were performed on a sample of clinically relevant predictors (table A and B). Binary predictors were selected instead of continuous due to the binary cut-offs used within the IMCI chart booklet and ePOCT+ algorithm. Predictors with no observations within the two by two table were omitted from the analysis. Predictors for which the lower and upper 95% confidence intervals (CI) of the odds ratio (OR) do not overlap with 1, and the positive likelihood ratio (PLR) is 5 or above, or the negative likelihood ratio (NLR) is 0.2 or below, were considered to be significant predictors.

To further understand the independent prognostic value of each predictor, a multivariate logistic regression model with LASSO penalty was performed. A multivariable logistic regression for the probability of clinical failure was fitted to the predictors included in the bivariate analysis for the ePOCT and ALMANACH data sets. A LASSO penalty was used for feature selection. LASSO favours sparse solutions by shrinking less important coefficients to zero according to a penalty, which is proportional to the sum of the absolute values of the coefficients. LASSO exclusions are marked as “LASSO-excluded” in table A and B. Some features appear several times in the bivariate analysis but are binarised at different thresholds (e.g. hemoglobin). To select the appropriate threshold for the multivariable analysis, a sequential feature selection was used, where a model for each available threshold was computed and compared according to the pseudo r-squared measures. The threshold that resulted in the best model was selected for inclusion into the final model. Excluded thresholds are marked as “redundant thresholds” in table A and B. The odds ratio (with 95% confidence interval) was calculated using a multivariable logistic regression model without penalty for each remaining predictor (It is not possible to use the coefficients of the regression with penalty).

The per protocol population was used for this analysis since the intention to treat population considered all patients lost to follow-up as clinical failure. All analyses were performed using Stata (version 16) and Python 3.9.

**Results and Discussion**

Clinical failure at day 7 occurred in 2.3% (37/1586) of children managed using ePOCT, and 4.1% (65/1573) of children managed using ALMANACH. The bivariate logistic regression model found ePOCT danger signs (Unconscious, lethargic, 2 or more convulsions, or convulsing now) OR 12.4 (95% CI 2.5, 62); ALMANACH danger signs OR 13.5 (95% CI 6.6, 27.6), chest indrawing (ePOCT OR 9.3 (95% CI 2.9, 30.2); ALMANACH OR 17.2 (95% CI 7, 42.1)), hypoxemia <90% (ePOCT OR 29.4 (95% CI 4.8, 181.8)), respiratory distress (ALMANACH OR 7 (95% CI 3.2, 15.3) severe general appearance (ePOCT OR 9.4 (95% CI 2.8, 31.6), somnolence (ALMANACH OR 6.9 (95% CI 1.4, 34.1), any sign of anemia (ALMANACH OR 6.9 (95% CI 2.9, 16.6), and mid-upper arm circumference (MUAC) <12.5cm (ePOCT OR 12.3 (95% CI 3.3, 46.1); ALMANACH <11.5cm OR 53 (95% CI 12.9; 218.2)) to be prognostic of clinical failure. The multivariate logistic regression with LASSO penalty found respiratory distress and severe general appearance within ePOCT, and chest in-drawing, any sign of anemia and MUAC <12.5cm within ALMANACH to be prognostic of clinical failure. There are some limitations to the interpretation of this analysis. Notably, the prognostic value of each variable must be considered within the context of the original model. If the original model (ePOCT or ALMANACH) used a specific predictor to trigger a specific treatment or referral, then the prognostic value will likely be underestimated. This was apparent when comparing the prognostic value of very low weight-for-age z-score which resulted in antibiotics and a referral within the ePOCT algorithm, and not in ALMANACH [2]. As such, no rule was used to include or omit a clinical element within ePOCT+ based on this analysis, but helps contextualize how the algorithm branches can be improved. Future analyses however could specifically look at how the current algorithm can be improved in terms of prognostic and diagnostic accuracy, and model efficiency.

**Table A: Bivariate and multivariate logistic regression model of clinical elements used in ePOCT to predict day 7 clinical failure**

| **Prognostic factor** | **Bivariate analysis** | | | | | **Multivariate analysis with LASSO penalty** |
| --- | --- | --- | --- | --- | --- | --- |
|  | **OR (95% CI)** | **PLR** | **NLR** | **Sensitivity** | **Specificity** | **OR (95% CI)** |
| **Binary variables** | | | | | |  |
| Female | 1.1 (0.6, 2.1) | 1.0 | 1.0 | 46% | 56% | 1.3 (0.7, 2.6) |
| Hb <10 g/dL | 1.9 (0.9, 3.9) | 1.3 | 0.7 | 70% | 45% | 1.9 (0.9, 4.1) |
| Hb <7 g/dL | 1.9 (0.6, 6.4) | 1.8 | 1.0 | 8% | 96% | Redundant threshold |
| Hb <6 g/dL | 2.2 (0.3, 17.2) | 2.2 | 1.0 | 3% | 99% | Redundant threshold |
| Hb <5 g/dL | 4.8 (0.6, 38.5) | 4.7 | 1.0 | 3% | 99% | Redundant threshold |
| Chest indrawing | **9.3 (2.9, 30.2)** | **8.1** | 0.9 | 14% | 98% | 2.2 (0.5, 9.9) |
| Respiratory distress | **4.7 (2.2, 10.1)** | 2.9 | 0.6 | 50% | 82% | **5.0 (2.3, 11.0)** |
| ePOCT Danger signs (unconscious, lethargic, >=2 convulsions or convulsing now) | **12.4 (2.5, 62)** | **11.8** | 0.9 | 6% | 100% | Not kept in analysis |
| Diarrhea | 0.9 (0.3, 2.4) | 0.9 | 1.0 | 14% | 85% | LASSO-excluded |
| Very low weight for age (<-3 WAZ) | 1.5 (0.3, 6.3) | 1.4 | 1.0 | 5% | 96% | LASSO-excluded |
| MUAC <12.5cm | **12.3 (3.3, 46.1)** | **11.2** | 0.9 | 10% | 99% | 2.1 (0.6, 7.9) |
| Hypoxemia <90% | **29.4 (4.8, 181.8)** | **27.9** | 0.9 | 5% | 100% | 7.0 (0.8, 59.7) |
| Hypoxemia <93% | 4.2 (0.9, 18.4) | 4.0 | 1.0 | 5% | 99% | Redundant threshold |
| Respiratory Rate >=50%ile | 1.5 (0.6, 3.8) | 1.1 | 0.7 | 86% | 19% | Redundant threshold |
| Respiratory Rate >=75%ile | 1.3 (0.7, 2.4) | 1.1 | 0.9 | 54% | 52% | Redundant threshold |
| Respiratory Rate >=90%ile | 1.6 (0.8, 3.1) | 1.4 | 0.9 | 32% | 76% | 1.3 (0.6, 2.8) |
| Respiratory Rate >=97%ile | 1.1 (0.3, 3.6) | 1.1 | 1.0 | 8% | 93% | Redundant threshold |
| Heart Rate >=50%ile | 0.8 (0.4, 1.9) | 0.9 | 1.0 | 22% | 75% | 0.7 (0.3, 1.6) |
| Heart Rate >=75%ile | 0.3 (0, 2.5) | 0.4 | 1.1 | 3% | 92% | Redundant threshold |
| General appearance (Normal, severe) | **9.4 (2.8, 31.6)** | 83.7 | 0.9 | 5% | 100% | **127.8 (10.7, 1525.7)** |

CI Confidence Interval; Hb hemoglobin; MUAC mid-upper arm circumference; NLR Negative Likelihood ratio; OR Odds ratio; PLR Positive Likelihood ratio; %ile percentile

**Table B: Bivariate and multivariate logistic regression model of clinical elements used in ALMANACH to predict day 7 clinical failure**

| **Prognostic factor** | **Bivariate analysis** | | | | | **Multivariate analysis with LASSO penalty** |
| --- | --- | --- | --- | --- | --- | --- |
|  | **OR (95% CI)** | **PLR** | **NLR** | **Sensitivity** | **Specificity** | **OR (95% CI)** |
| **Binary variables** | | | | | |  |
| Female | 1.3 (0.8, 2.1) | 1.1 | 0.9 | 51% | 55% | 1.2 (0.7, 2.0) |
| Danger sign (History of convulsions, unable to drink, unconscious/lethargic, vomiting everything, jaundice, cyanosis, stiff neck, severe pallor, severe wasting) | **13.5 (6.6, 27.6)** | **11.0** | 0.8 | 20% | 98% | 2.8 (0.7, 11.3) |
| Any sign of dehydration | **4.2 (1.6, 11.2)** | 3.9 | 0.9 | 8% | 98% | 2.1 (0.6, 7.3) |
| Sunken eyes | 3.8 (0.6, 24.8) | 2.7 | 0.7 | 40% | 85% | LASSO-excluded |
| Chest indrawing | **17.2 (7, 42.1)** | **14.0** | 0.8 | 20% | 99% | **6.1 (1.2, 32.1)** |
| Respiratory distress | **7 (3.2, 15.3)** | **5.8** | 0.8 | 20% | 97% | LASSO-excluded |
| Somnolence | **6.9 (1.4, 34.1)** | **6.8** | 1.0 | 3% | 100% | LASSO-excluded |
| Diarrhea | 1.1 (0.6, 2.1) | 1.1 | 1.0 | 21% | 81% | LASSO-excluded |
| Very low weight for age (<-3 WAZ) | **3.7 (1.5, 9.1)** | 3.5 | 0.9 | 9% | 97% | 0.3 (0.1, 1.2) |
| Respiratory Rate >=50%ile | 1.3 (0.6, 2.6) | 1.0 | 0.8 | 85% | 19% | Redundant threshold |
| Respiratory Rate >=75%ile | 1.7 (1, 2.8) | 1.3 | 0.7 | 63% | 50% | 1.4 (0.8, 2.5) |
| Respiratory Rate >=90%ile | 1.6 (1, 2.7) | 1.4 | 0.9 | 35% | 75% | Redundant threshold |
| Respiratory Rate >=97%ile | 1.3 (0.6, 2.7) | 1.2 | 1.0 | 12% | 90% | Redundant threshold |
| Heart Rate >=50%ile | 1.5 (0.8, 2.6) | 1.3 | 0.9 | 27% | 79% | Redundant threshold |
| Heart Rate >=75%ile | 1.7 (0.7, 4.1) | 1.6 | 1.0 | 10% | 94% | 1.3 (0.5, 3.5) |
| Any otitis incl discharge | 0.8 (0.1, 6) | 0.8 | 1.0 | 2% | 98% | LASSO-excluded |
| Any skin infection incl severe | 0.5 (0.1, 2.3) | 0.6 | 1.0 | 3% | 95% | 0.4 (0.1, 2.1) |
| Any sign of anemia | **6.9 (2.9, 16.6)** | **6.3** | 0.9 | 11% | 98% | **3.6 (1.2, 10.7)** |
| MUAC <11.5cm | **53 (12.9, 218.2)** | **47.5** | 0.9 | 11% | 100% | Redundant threshold |
| MUAC <12.5cm | 2.5 (0.3, 20.1) | 2.5 | 1.0 | 2% | 97% | **12.6 (5.0, 31.4)** |

CI Confidence Interval; Hb hemoglobin; MUAC mid-upper arm circumference; NLR Negative Likelihood ratio; OR Odds ratio; PLR Positive Likelihood ratio; %ile percentile

**References**

1. Keitel K, Kagoro F, Samaka J, Masimba J, Said Z, Temba H, et al. A novel electronic algorithm using host biomarker point-of-care tests for the management of febrile illnesses in Tanzanian children (e-POCT): A randomized, controlled non-inferiority trial. PLoS medicine. 2017;14(10):e1002411. Epub 2017/10/24. doi: 10.1371/journal.pmed.1002411. PubMed PMID: 29059253; PubMed Central PMCID: PMCPMC5653205.

2. Tan R, Kagoro F, Levine GA, Masimba J, Samaka J, Sangu W, et al. Clinical Outcome of Febrile Tanzanian Children with Severe Malnutrition Using Anthropometry in Comparison to Clinical Signs. American Journal of Tropical Medicine and Hygiene. 2020;102(2):427-35. doi: 10.4269/ajtmh.19-0553. PubMed PMID: WOS:000512881500035.
