# Supplementary material for: ePOCT+ and the medAL-suite: Development of an electronic clinical decision support algorithm and digital platform for pediatric outpatients in low- and middle-income countries
Source: PLOS Digit Health. 2023 Jan 19;2(1):e0000170. doi: 10.1371/journal.pdig.0000170 (PMC9931356; doi:10.1371/journal.pdig.0000170)
Supplement: S4 Appendix — (DOCX) [file pdig.0000170.s004.docx]

**S4 Appendix: Features of the medAL-*creator* and medAL-*reader software as defined by a clinical-IT collaboration with end-user feedback***

| **Programming eCDSA platform (medAL-*creator*)** | |
| --- | --- |
| **Feature** | **Description / rationale / example** |
| Easy platform so that clinician can program and/or review the algorithm | Drag and drop interface, obvious connectors, no visible scripts |
| Allow the integration of Weighted and Boolean algorithms to reach a diagnosis | Boolean (and, or, not);  Weighted algorithm; Based on a score using weighted variables |
| Allow for inclusion of sub-algorithms in any algorithm (predefined syndromes) | Ease the maintenance (and reduce risks of errors) for predefined syndromes that appear in several algorithms |
| Allow a diagnosis to exclude another diagnosis | Severe or complicated diagnoses can exclude non-severe and uncomplicated diagnoses |
| Allow a management to exclude other managements | Ex. Guidance to refer a patient to the hospital for one diagnosis excludes ‘no referral’ from another diagnosis |
| Allow a drug to exclude another drug | Ex. A broad spectrum antibiotic could exclude another narrow spectrum antibiotic |
| Ability to make a variable/question mandatory or not mandatory to respond | Allowing users the ability to skip non-essential questions to speed up processes |
| Allow the use of reference tables for clinical signs | In order to calculate z-scores and percentiles |
| Allow for cross-referencing of variables | To compute the BMI based on the weight and height of the patient, for instance |
| Allow conditioning of variables within the decision tree algorithm and for individual variables using specific filters | Variables only appear based on previous responses based on the decision tree logic, and based on certain filters (complaint categories) |
| Allow for management of multiple versions of different algorithms | Each version can be deployed to different users |
| Generation of data dictionary | Allow for future integration with alternative variable nomenclature (SNOMED, CID) |
| Algorithm validation mechanisms | An automatic validation process identifies errors in decision tree logic before an algorithm can be deployed |
| Modification restrictions to deployed algorithms | Restrictions to modify decision logic for algorithms that are implemented and in use (only minor modifications possible). New versions, however can make modifications and deployed allowing for HCW to understand the changes made. |
| Automatic conversion of the algorithm into a machine-readable code | Transforming “human-readable” drag and drop decision tree into machine-readable code for execution on the medAL-*reader* application |
| **eCDSA platform (medAL-*reader*)** | |
| **Feature** | **Description / rationale / example** |
| Multi-modal use on local network | Allow the use of different users, on different devices to manage a single patient |
| Ability for clinician using eCDSA to perform multiple, simultaneous consultations, with pause and resume capability | Allowing a health care provider to see another patient while sending another patient for laboratory investigations |
| Ability to accept, refuse, and add diagnoses and treatments proposed by the algorithm | To improve algorithms, monitor quality of care, and provide dosing for drugs not proposed by the algorithm. |
| To follow natural flow of consultation | First excluding emergency signs, evaluating the chief complaints, medical history, physical exam, investigations (laboratory tests), Diagnosis, treatment and management. |
| Option for user to move forward and backwards through the consultation process | To be able to update information from other stages if they are provided at a later stage |
| Follow-up questions/variables conditioned by root variables | Ex. Duration of cough, only to appear if cough present |
| Access to emergency management via an emergency button at any point during the consultation, even if for a different patient. | Without an emergency button to be able to press at any moment, a clinician will not be given immediate guidance for emergencies. |
| Alerts when clinician selects an emergency or danger sign | To be able to provide emergency management guidance if needed |
| Outline variables that would result in a referral | To motivate clinicians to assess danger signs with additional precaution |
| Division of medical history questions and physical exam signs by system | Organize consultation flow as clinicians are trained to; ie by system |
| Warning and error limit messages should advise clinicians of continuous values that are out of normal range, and out of feasible range. | To assure safety and quality of data inputted. |
| Provide option to give information and photos about each variable, diagnosis, drug and management. | Info buttons are placed beside each variable, diagnosis and management so that the clinician can get more information.  The images will be notably helpful for helping diagnose skin rashes and other physical signs. |
| Ability for user to state that some medical history questions are “unknown”, some physical exams or anthropometric measurements are “not feasible” to measure, and some tests are not available. | In order to prevent the user from being blocked from continuing the assessment and prevent the input of false data. |
| Alert for user if they have not answered an important/mandatory question | To assure that clinically relevant and important questions are answered |
| A case summary is provided at the end of the consultation with the most urgent and important diagnoses listed first | To have a short summary of the previous consultation when the patient comes back for a follow-up or new visit |
| Ability to retrieve patient information from previous consultations using patient registration information | To facilitate follow-up consultations |
| Calculate medication dosing according to weight, age and formulation | Reduce error in medication dosing. |
| Support for translation | Allow for use of the same clinical algorithm in different languages |
| Have online and offline capacity | Health care workers should be able to use the tool online/offline |
| Data collection and synchronization to a central server | Through secure (encrypted) channels |
| Destination of the data from the app must be configurable | In order to comply with national regulators |
