# Supplementary material for: ePOCT+ and the medAL-suite: Development of an electronic clinical decision support algorithm and digital platform for pediatric outpatients in low- and middle-income countries
Source: PLOS Digit Health. 2023 Jan 19;2(1):e0000170. doi: 10.1371/journal.pdig.0000170 (PMC9931356; doi:10.1371/journal.pdig.0000170)
Supplement: S5 Appendix — (DOCX) [file pdig.0000170.s005.docx]

**S5 Appendix: Evaluation of ePOCT+ based on the characteristics set by the target product profile for electronic clinical decision support algorithm as defined by expert consensus[1]**

| **General scope** | | |
| --- | --- | --- |
| **Characteristics** | **Minimal / Optimal requirements** | **ePOCT+ / medAL-reader** |
| Intended use | Optimal | Captures diagnostic test results, patient clinical data to provide treatment and care recommendations |
| Target population | Optimal | Defined target population. Inclusion and exclusion criteria used when enrolling the patient |
| Setting | Optimal | Different algorithms used for different countries |
| Targeted end user | Optimal | Algorithms designed for use by nurses, physician assistants, but can also be used by medical doctors |
| Algorithm access | Optimal | medAL-*reader* app can be downloaded on android based devices |
| Algorithm content | Optimal and Planned | Based on WHO/international/local clinical care guidelines, peer-reviewed articles, and clinical experience/practice and clinical validation research.[2-7] For new algorithms clinical validation is planned in the form of cluster randomized trials. |
| Algorithm treatment recommendations | Optimal | Treatment recommendation based on international and national treatment guidelines, prioritizing medications available at the lowest level of care. Dosing calculated for clinicians. Treatment recommendations support antimicrobial stewardship. |
| Compatible POC tools | Optimal | POC tools used in routine care, and emerging diagnostic tools and devices relevant to the algorithm are included (CRP, Pulse oximetry) |
| Regulated toolkit components | Minimal | POC diagnostic tests and medical devices are regulatory approved, compliant with local regulations, and in the case of Tanzania included in the Standard medical laboratory equipment to be used at dispensary and health centre level (CRP, pulse oximetry, hemoglobin, HIV, malaria, syphilis, glucose)[8] |
| Compatible devices | Optimal | App is compatible with large smartphones, and tablets. (Compatible with computers through tablet mirroring) |
| Compatible operating systems | Optimal | medAL-*reader* compatible with android devices |
| **Clinical decision support algorithm** | | |
| **Characteristics** | **Minimal / Optimal requirements** | **ePOCT+ / medAL-reader** |
| Content transparency | Optimal | The healthcare programme and end user have access to underlying evidence and methodology used to develop the algorithm |
| Quality control | Optimal | The algorithm underwent both analytical and semantical verification. |
| Algorithm validation | Planned | While many of the algorithms have previously been validated, new content will be validated through a cluster randomized controlled trial |
| Machine learning | Planned | Machine learning models were used to help validated the use of certain predictors in the algorithms.  Following validation of the static ePOCT+ algorithms, machine learning models will be used to improve the algorithms, and validated in randomized controlled trials |
| POC data input | Optimal | POC data can be inputted in ePOCT+ / medAL-reader |
| Disease likelihood (POC tool) | Optimal | Prognostic positive/negative likelihood ration and pretest probability evaluated for all POC predictors (including Hemoglobin, glucose, pulse oximetry) in children presenting with fever from the community.[9]  CRP: Based on Diagnostic positive/negative likelihood ratio and pretest probability from the setting of interest.[10] Also evaluated in randomized controlled trial.[3] |
| POC training | Optimal | Training was provided to all end-users for all new POC tests/tools not normally used in routine care |
| **App** | | |
| **Characteristics** | **Minimal / Optimal requirements** | **medAL-*creator* and medAL-*reader*** |
| System validation | Optimal* | - Valid clinical association *And* clinical validation: Supported by well-established or novel evidence.  *Cluster randomized trials will be conducted to assure validity for algorithms without established evidence.  - Analytical validation: Multiple pathways for all algorithms were tested to assure that inputted data is processed correctly into expected output |
| System access | Minimal | Data access protected by authentication and authorization. |
| Context configuration | Optimal | Translation possible, country preferences for the algorithm can be configurable |
| Customisation | Optimal | Algorithms can be modified using medAL-*creator* including to updates to the list of medicines and POCs. |
| User access rights | Optimal | Roles can be assigned to provide different levels of data access |
| Expert support | No | Access to online/remote expert advice to assist in patient consultation is not possible |
| App training | Minimal | On-site training |
| Internet availability | Optimal | Works offline and can trigger alerts for synchronization |
| Clinical data entry | Minimal | Manual entry by the operator |
| Patient management recommendation | Minimal | Consultation data summarized and actionable recommendations provided. Interoperability with EMRs and HIS is planned. |
| Navigation | Optimal | Non-sequential: the user can move to a certain extent in any direction through an assessment and change input data to reach a final recommendation |
| Workflow requirements to enable time-delayed POC data input | Minimal | User can perform multiple, simultaneous consultations, with pause and resume capability, to allow clinical and laboratory data entry |
| Task management | Optimal | Multiple algorithms can be supported simultaneously in one application against a common data set |
| Follow-up | Optimal | Ability to retrieve patient information using patient registration information. However data from previous consultations cannot be automatically integrated within the algorithms for the new follow-up consultation |
| System malfunction protection | Optimal | System malfunctions are made clear to the user |
| Scalability | Optimal | The app allows for high transaction volumes with complex workflows to cover primary care workforce at a national scale |
| Updates and versioning | Optimal | Processes are in place to control any app changes (including algorithm version updates) and provide the appropriate and correct update to the user |
| **Data** | | |
| **Characteristics** | **Minimal / Optimal requirements** | **medAL-*reader*** |
| Data capture | Optimal* | Can capture text, image, numeric, GPS, barcode.  Does not capture audio, video or biometric |
| Data validation | Optimal | The warning and error alerts can be programmed to prevent errors of data input |
| Data ownership | Optimal | The healthcare programme of the country of implementation has ownership of the data |
| Data storage | Optimal | The healthcare programme can choose the destination of the app’s data |
| Data recovery | Optimal | The system can be re-established to the desired state in the event of interruption or failure. Data is saved upon completion of each stage (registration, 1^st^ assessment, medical history and physical exam, tests, and diagnosis and management.) |
| Data flow | Optimal | The flow of data is determined by the healthcare programme |
| Data reporting | Optimal | Dashboards will be configured to present real-time data for reporting, benchmarking and monitoring |
| Data provenance | Optimal | Provides origin and processes applied to output data. When data are downloaded or shared, the version of the model is tagged so it is always clear how the data was obtained |
| Data dictionary | Planned | Data dictionary is automatically created by medAL-*creator*. Link to international reference standard terminology in development. |
| Data security and privacy | Optimal | The app operates under secure connectivity which meets data protection  and regulations of individual countries to avoid loss and corruption of  sensitive data, and mitigate cyberattacks, whether data are at rest or in  transmission.  Includes:  ► Authorisation/access control  ► De-identified data  ► Data encryption  ►Two-factor authentication |

CRP, C-reactive protein; GPS, Global Positioning System; POC, point-of-care; WHO, World Health Organization

**References**

1. Pellé KG, Rambaud-Althaus C, Acremont V, Moran G, Sampath R, Katz Z, et al. Electronic clinical decision support algorithms incorporating point-of-care diagnostic tests in low-resource settings: a target product profile. BMJ Global Health. 2020;5(2):e002067. doi: 10.1136/bmjgh-2019-002067.

2. Keitel K, Kagoro F, Samaka J, Masimba J, Said Z, Temba H, et al. A novel electronic algorithm using host biomarker point-of-care tests for the management of febrile illnesses in Tanzanian children (e-POCT): A randomized, controlled non-inferiority trial. PLoS medicine. 2017;14(10):e1002411. Epub 2017/10/24. doi: 10.1371/journal.pmed.1002411. PubMed PMID: 29059253; PubMed Central PMCID: PMCPMC5653205.

3. Keitel K, Samaka J, Masimba J, Temba H, Said Z, Kagoro F, et al. Safety and Efficacy of C-reactive Protein–guided Antibiotic Use to Treat Acute Respiratory Infections in Tanzanian Children: A Planned Subgroup Analysis of a Randomized Controlled Noninferiority Trial Evaluating a Novel Electronic Clinical Decision Algorithm (ePOCT). Clinical Infectious Diseases. 2019;69(11):1926-34. doi: 10.1093/cid/ciz080.

4. Tan R, Kagoro F, Levine GA, Masimba J, Samaka J, Sangu W, et al. Clinical Outcome of Febrile Tanzanian Children with Severe Malnutrition Using Anthropometry in Comparison to Clinical Signs. American Journal of Tropical Medicine and Hygiene. 2020;102(2):427-35. doi: 10.4269/ajtmh.19-0553. PubMed PMID: WOS:000512881500035.

5. Rambaud-Althaus C, Shao A, Samaka J, Swai N, Perri S, Kahama-Maro J, et al. Performance of Health Workers Using an Electronic Algorithm for the Management of Childhood Illness in Tanzania: A Pilot Implementation Study. The American journal of tropical medicine and hygiene. 2017;96(1):249-57. Epub 2017/01/13. doi: 10.4269/ajtmh.15-0395. PubMed PMID: 28077751; PubMed Central PMCID: PMCPMC5239703.

6. Shao AF, Rambaud-Althaus C, Samaka J, Faustine AF, Perri-Moore S, Swai N, et al. New Algorithm for Managing Childhood Illness Using Mobile Technology (ALMANACH): A Controlled Non-Inferiority Study on Clinical Outcome and Antibiotic Use in Tanzania. PLoS One. 2015;10(7):e0132316. Epub 2015/07/15. doi: 10.1371/journal.pone.0132316. PubMed PMID: 26161535; PubMed Central PMCID: PMCPMC4498627.

7. Shao AF, Rambaud-Althaus C, Swai N, Kahama-Maro J, Genton B, D'Acremont V, et al. Can smartphones and tablets improve the management of childhood illness in Tanzania? A qualitative study from a primary health care worker's perspective. BMC health services research. 2015;15:135-. doi: 10.1186/s12913-015-0805-4. PubMed PMID: 25890078.

8. Tanzania Ministry of Health CD, Gender, Elderly and Children. Standard Medical Laboratory Equipment Guideline (SMLEG). Tanzania2018.

9. Chandna A, Tan R, Carter M, Van Den Bruel A, Verbakel J, Koshiaris C, et al. Predictors of disease severity in children presenting from the community with febrile illnesses: a systematic review of prognostic studies. BMJ Glob Health. 2021;6(1). Epub 2021/01/22. doi: 10.1136/bmjgh-2020-003451. PubMed PMID: 33472837.

10. Erdman LK, D'Acremont V, Hayford K, Rajwans N, Kilowoko M, Kyungu E, et al. Biomarkers of Host Response Predict Primary End-Point Radiological Pneumonia in Tanzanian Children with Clinical Pneumonia: A Prospective Cohort Study. PLoS One. 2015;10(9):e0137592. Epub 2015/09/15. doi: 10.1371/journal.pone.0137592. PubMed PMID: 26366571; PubMed Central PMCID: PMCPMC4569067.
