# Supplementary material for: ePOCT+ and the medAL-suite: Development of an electronic clinical decision support algorithm and digital platform for pediatric outpatients in low- and middle-income countries
Source: PLOS Digit Health. 2023 Jan 19;2(1):e0000170. doi: 10.1371/journal.pdig.0000170 (PMC9931356; doi:10.1371/journal.pdig.0000170)
Supplement: Attachment — Submitted filename: Response to reviewers.doc [file pdig.0000170.s006.doc]

| Ryan S McGinnis,  Academic Editor  PLOS Digital Health | Rainer Tan  Rainer.Tan@unisante.ch  +255 676 020 505 |
| --- | --- |
|  | Tanzania, 20 September 2022 |

**RE: Response to reviewers for manuscript: PDIG-D-22-00055: Development of ePOCT+ and the medAL-*suite***

Dear Dr McGinnis,

Thank you for inviting us to submit a revised version of the manuscript.

Below are our responses to the different points raised during the review process.

Note all reference to line numbers are the lines within the track changes document using the “All Markup” view.

A) **Journal Requirements**

1. **Please ensure that the funders and grant numbers match between the Financial Disclosure field and the Funding Information tab in your submission form. Note that the funders must be provided in the same order in both places as well.**

We confirm that the funders and grant numbers match.

**2. Please update your online Competing Interests statement. If you have no competing interests to declare, please state: “The authors have declared that no competing interests exist.”**

The competing interest statement has been updated

**3. In the online submission form, you indicated that your data will be submitted to a repository upon acceptance. We strongly recommend all authors deposit their data before acceptance, as the process can be lengthy and hold up publication timelines. Please note that, though access restrictions are acceptable now, your entire data will need to be made freely accessible if your manuscript is accepted for publication. This policy applies to all data except where public deposition would breach compliance with the protocol approved by your research ethics board. If you are unable to adhere to our open data policy, please kindly revise your statement to explain your reasoning and we will seek the editor's input on an exemption. Please be assured that, once you have provided your new statement, the assessment of your exemption will not hold up the peer review process.**

The dataset is available on Zenodo; DOI: 10.5281/zenodo.400380 we have updated the online submission form.

**4. Please ensure that the Title in your manuscript and the Title in your online submission form are the same.**

We confirm that the Title in our manuscript is the same as the online submission form.

**5. Please provide separate figure files in .tif or .eps format only and remove any figures embedded in your manuscript file. Please also ensure that all files are under our size limit of 10MB.**

We have removed all figures embedded in the manuscript file, and uploaded them separately in the .tif format.

**6. Some material included in your submission may be copyrighted. According to PLOS’s copyright policy, authors who use figures or other material (e.g., graphics, clipart, maps) from another author or copyright holder must demonstrate or obtain permission to publish this material under the Creative Commons Attribution 4.0 International (CC BY 4.0) License used by PLOS journals. Please closely review the details of PLOS’s copyright requirements here: PLOS Licenses and Copyright. If you need to request permissions from a copyright holder, you may use PLOS's Copyright Content Permission form.

Please respond directly to this email or email the journal office and provide any known details concerning your material's license terms and permissions required for reuse, even if you have not yet obtained copyright permissions or are unsure of your material's copyright compatibility.

Potential Copyright Issues:

Figures 1 and 2: Please confirm whether you drew the images / clip-art within the figure panels by hand. If you did not draw the images, please provide (a) a link to the source of the images or icons and their license / terms of use; or (b) written permission from the copyright holder to publish the images or icons under our CC-BY 4.0 license. Alternatively, you may replace the images with open source alternatives. See these open source resources you may use to replace images / clip-art:
- https://commons.wikimedia.org
-** [**https://openclipart.org/**](https://openclipart.org/)

Figures 1 and 2 have now been re-drawn in order to only contain clip-art from openclipart.org or drawn by us.

**7. Please review your reference list to ensure that it is complete and correct. If you have cited papers that have been retracted, please include the rationale for doing so in the manuscript text, or remove these references and replace them with relevant current references. Any changes to the reference list should be mentioned in the rebuttal letter that accompanies your revised manuscript. If you need to cite a retracted article, indicate the article's retracted status in the References list and also include a citation and full reference for the retraction notice.**

We have reviewed the reference list and confirm that it is complete and correct

B) **Reviewer 1 comments**

**This is a potentially useful paper for individuals interested in developing clinical decision support algorithms and other CDS tools. The general process of development is good, but some areas need more explanation to be useful to others. These areas are described below.**

We thank reviewer 1 for their helpful comments.

**B1) lines 178-180-- If a number of key variables are omitted how does that affect the validity of the algorithm? This needs more discussion**

This is a good point, in fact the omission of these variables are in line with the WHO IMCI strategy that also excludes these variables within their protocols. As such they are not true omissions, but rather they are clinical variables that were not added. As such it does not affect the validity of the algorithm.

We have modified the text in line 182 to replace “to the omission of” with “*to us not adding”,* and added the text in line 184 *“Importantly, these symptoms and signs are also not included within IMCI, likely for similar reasons.”*

**B2) line 268-- Describe the more formal decision processes that were used.**

We agree that more detail would be beneficial. We have added the following text in line 275:

*“In Tanzania and Rwanda a modified nominal group method was used, in which each participant one-by-one provided their opinion on the presented branch of the algorithm, followed by a group discussion and then an absolute majority vote for the final version.”*

**B3) lines 314-318-- More detail on the field studies and user testing experience should be included.**

We have now modified the paragraph in question: lines 323-331 now include more details on the different tests.

We provide further description of the feasibility tests in the results section, lines 359 – 362 and table 1. Within table 1 we describe some of the most relevant and interesting feedback and observations, we do not feel there are other observations of relevance to be added. If the editor agrees we would refrain from adding more detail on other feedback given that were often quite minor, or of less relevance in regards to the scope of this manuscript.

C) **Reviewer 2 comments**

**Thank you for the opportunity to review this manuscript, which describes development of a new generation CDSA ePOCT, with expanded age groups and perceived user-friendly interface, that has been implemented in a number of countries, and that is awaiting further validation in clinical studies.

The manuscript is generally well written, albeit repetitive in some areas. The authors describe in detail the background process undertaken to improve the content of the CDSA, which was comprehensive and for which they should be congratulated, and they include a description of the feedback provided from users which was taken on to further improve the tool.**

We thank reviewer 2 for their helpful comments.

**The following are some comments for the authors to consider regarding their manuscript -

Major -
c1) Abstract:
Suggest that it is important to highlight in the abstract that the algorithm is undergoing further validation in clinical studies.**

We agree and have added the following sentence to the abstract:

*“Further validation clinical studies are underway in Tanzania, Rwanda, Senegal, Kenya, and India. “*

**Clinical Algorithm:
C2) line 158 - authors state that ‘for conditions not covered in these guidelines’ - is it not clear what the process/reference was for identifying conditions not covered in these guidelines, could the authors please expand, so that readers understand the systematic approach taken, or is this entire paragraph repeating from paragraph commencing line 145 on page 7?**

Thank you for this comment. Indeed it was not clear, this section was describing what sources were used when the national guidelines did not provide sufficient information. We have reworded that section to make this more clear:

*“If there was not sufficient detail in order to derive decision logic from these national guidelines, a brief review of literature was conducted to identify peer-reviewed literature and other international guidelines.”*

Of note, we also comment about this process in the discussion line 382:

“Often supplementary evidence was needed to complement national and international guidelines. This evidence should ideally be identified through systematic reviews,[59] however those are not always feasible. Leveraging existing evidence databases as done by another CDSA may be a more feasible method to avoid biases in identifying supporting evidence.[60]”

**Feasibility, acceptability, and reliability of predictors:
C3) The authors refer to the Delphi survey amongst 30 Tanzanian healthcare workers which is further described in the appendix, that lead to omission of widely recognised warning signs e.g. capillary refill time and cool peripheries — could authors consider commenting, perhaps in the discussion - about whether these signs are definitely not feasible in all settings that the CDSA is currently being implemented, and speculate on whether appropriate training should be implemented / what difference this would make to morbidity and mortality, based on other studies in similar settings (i.e. other widely implemented CDSAs)? Based on the author’s previous studies referenced in the supplementary appendix comparing ALMANACH with ePOCT, perhaps these signs are not highly important but I think it would add depth to the paper to discuss this element.**

This is a very good point, we have already adapted the text (see Question B1 from reviewer 1), and have added a paragraph to comment on this. Line 391:

“A number of considerations were taken into account when digitalizing and adapting paper guidelines. Among the most important considerations were the feasibility, acceptability, reliability, and diagnostic and prognostic performance of individual clinical elements, while also considering the overall performance of the algorithms in relation to the pre-test probability of the outcome or disease, and the clinician’s overall impression. Often conflicts can arise among the various factors that must be considered, which leads to difficult decisions. For example the Delphi survey among Tanzanian health care workers found that capillary refill time may not be feasible in primary health settings, however it has been found to have good prognostic value.[34] Such difficult decisions were often taken with input from clinical experts from the country of implementation. Additional training on clinical signs deemed not feasible, could potentially allow for future modifications.”

**C4) Furthermore, could authors further describe or state the number of feasibility tests done on real and fictional cases that led to this omission in this section of the manuscript or supplementary appendix?**

See answers to reviewer 1, question B3, we have provide more detail on the feasibility tests. The rationale for omissions are addressed in questions C3. The number of fictional cases were added to line 323, the number of feasibility tests on real cases is outlined in line 359.

**C5) In the feasibility tests, authors do not comment on the training required to implement this CDSA in the target setting - i.e. how much time is required for primary healthcare workers to be trained to use this tool?**

We have now clarified that 2 days of training took place before feasibility tests: line 359

**C6) Table 1 - could authors consider expanding on the decision for some clinical signs that were not measured to be allowed to be an estimate rather than (or in addition to) a “not measured” option?**

We have provided further discussion on this point in the discussion line 400:

*“*Another difficult decision included the option of estimating results when measurements are not possible (e,g, respiratory rate). Health care workers often do not measure respiratory rate when following paper guidelines or using a CDSA.[6, 18] If the CDSA does not allow the option of not being able to measure respiratory rate then they may not be able to move forward using the tool, or may enter false data if indeed respiratory rate measurement is not feasible. Allowing health care workers to estimate the value is not ideal, but allows the health care worker to at the very least visually assess respiratory rate, and provide an input in order for the algorithm to reach a diagnosis. This data can then be used to mentor health care workers that do not measure respiratory rate. Allowing clinicians to simply enter that respiratory rate was not possible without estimating, could be an option to consider, but would complicate the decision on what diagnosis to reach when selecting this option.”

**Discussion:
Would the authors like to comment on how their CDSA compares to other widely used CDSAs in this setting if applicable**

We are currently not in a position to be able to adequately compare the performance of ePOCT+ with other CDSAs, we hope to do this once the results of our quantitative and qualitative studies are completed.

**Minor -
Figure 3 is difficult to read - font small and blurry in centre algorithm**

Figure 3 has now been modified.

D) **Additional grammatical corrections/edits**

If the editor accepts upon re-reading the manuscript we have proposed to make minor grammatical corrections that does not change the general intention of the manuscript. They are all included in the track changes version.
