# Supplementary material for: ePOCT+ and the medAL-suite: Development of an electronic clinical decision support algorithm and digital platform for pediatric outpatients in low- and middle-income countries
Source: PLOS Digit Health. 2023 Jan 19;2(1):e0000170. doi: 10.1371/journal.pdig.0000170 (PMC9931356; doi:10.1371/journal.pdig.0000170)
Supplement: Attachment — Submitted filename: Response to reviewers2.doc [file pdig.0000170.s007.doc]

| Ryan S McGinnis,  Academic Editor  PLOS Digital Health | Rainer Tan  Rainer.Tan@unisante.ch  +41 76 269 6193 |
| --- | --- |
|  | Lausanne, 14 October 2022 |

**RE: 2^nd^ Response to reviewers for manuscript: PDIG-D-22-00055: Development of ePOCT+ and the medAL-*suite***

Dear Dr McGinnis,

Thank you for inviting us to submit a revised version of the manuscript.

Below are our responses to the different points raised during the review process.

Note all reference to line numbers are the lines within the track changes document using the “All Markup” view.

A) **Journal Requirements**

1. **Please review your reference list to ensure that it is complete and correct. If you have cited papers that have been retracted, please include the rationale for doing so in the manuscript text, or remove these references and replace them with relevant current references. Any changes to the reference list should be mentioned in the rebuttal letter that accompanies your revised manuscript. If you need to cite a retracted article, indicate the article's retracted status in the References list and also include a citation and full reference for the retraction notice.**

We confirm that the reference list is complete and correct, and do not cite papers that have been retracted.

The following references were modified or added:

- Correction; previous version was the “in press” reference:
  - Fink G, D'Acremont V, Leslie HH, Cohen J. Antibiotic exposure among children younger than 5 years in low-income and middle-income countries: a cross-sectional study of nationally representative facility-based and household-based surveys. The Lancet Infectious Diseases. 2020;20(2):179-87. doi: 10.1016/S1473-3099(19)30572-9.
- Added (more recent and relevant references):
  - Levine G, Bielicki J, Fink G. Cumulative Antibiotic Exposure in the First Five Years of Life: Estimates for 45 Low- and Middle-income Countries from Demographic and Health Survey Data. Clinical Infectious Diseases. 2022:ciac225. doi: 10.1093/cid/ciac225.
  - Chandna A, Osborn J, Bassat Q, Bell D, Burza S, D'Acremont V, et al. Anticipating the future: prognostic tools as a complementary strategy to improve care for patients with febrile illnesses in resource-limited settings. BMJ Glob Health. 2021;6(7). Epub 2021/08/01. doi: 10.1136/bmjgh-2021-006057. PubMed PMID: 34330761; PubMed Central PMCID: PMCPMC8327814.

**2. Figures 1-3 contains screenshots. We are not permitted to publish these under our CC-BY 4.0 license; websites are usually intellectual property and are copyrighted.This includes peripheral graphics of the web browser such as the buttons. We ask that you please remove or replace it.**

We confirm that figure 1 and 2 are not screenshots and comply with PLOS DH requirements, notably the figures only contain images / clip-art that we drew, or used open source images as outlined in the first response letter.

Figure 3 indeed contains screenshots, however they are screenshots of the graphic user interface of our web application (medal-creator) and a screenshot of the graphic user interface of our mobile application (medAL-reader), which we have designed, created, and released under the terms of the open-source MIT license. The mouse icon in the figure is a clip art open source image that we added to the screenshot, otherwise there are no other visible elements of any other graphic user interface (such as that of a browser, for instance).

Additional licensing information of both tools highlighted in the figure can be found here:

- medAL-*creator* license information: <https://github.com/Wavemind/liwi-backend/blob/develop/LICENSE>
- medAL-*reader* license information: <https://github.com/Wavemind/liwi-medal-reader/blob/main/LICENSE>

We hope this complies with your CC-BY 4.0 license, and happy to further discuss what we can do to be able to share this figure.

B) **Reviewer 1 comments**

**Most changes have been made, but a few still need to be addressed.  I could not find the number of feasibility tests on real cases in the body of the manuscript.  It did not seem to be where the authors said it was.**

We have clarified the number of feasibility tests in line 352:

*“*The feasibility tests of ePOCT+ were conducted in over 200 patients in 20 health facilities, leading to numerous modifications (Table 1). The improved algorithm was then piloted with over 2000 consultations following 2 days of training and on-site support, before officially starting the clinical validation studies in the five countries of implementation.”

**In addition, the authors were asked to compare their system to other similar systems.  Although it is too early for the actual results/performance to be compared, the design, intent, content of the system can and should be compared to other similar systems.**

We have added the following text to describe differences of ePOCT+ and medAL-reader compared to other CDSAs:

Line 361:

*“Novel content in the algorithm compared to other CDSA include decision logic for young infants less than 2 months, and in some countries decision logic for children 5-15 years old, and expanded clinical content for diagnoses not included in IMCI.”*

Line 369:

*“The development of medAL-creator, allows non-IT specialists to be able to program the clinical algorithms using a no-code, drag and drop interface, a novel solution that democratizes the development of CDSAs. This is a big advantage when compared to other CDSA tools that generally require advanced IT knowledge to review and program the code of the CDSA.”*

Line 413:

*“Compared to other CDSAs medAL-reader includes new functions such as an emergency button, and the ability to accept or refuse a diagnosis or treatment. The introduction of other digital tools such as electronic medical records within the same health facilities creates challenges in uptake and may result in duplication of processes.”*
